# A Compact Control System to Enable Automated Operation of Microfluidic Bioanalytical Assays

**DOI:** 10.3390/bios12121160

**Published:** 2022-12-13

**Authors:** Alan M. Gonzalez-Suarez, Alexander Long, XuHai Huang, Alexander Revzin

**Affiliations:** 1 Department of Physiology and Biomedical Engineering, Mayo Clinic, Rochester, MN 55905, USA; 2 Department of Biology, St. Olaf College, Northfield, MN 55057, USA

**Keywords:** microfluidics, automation, micromechanical valves

## Abstract

We describe a control system for operating valve-enabled microfluidic devices and leverage this control system to carry out a complex workflow of plasma separation from 8 μL of whole blood followed by on-chip mixing of plasma with assay reagents for biomarker detection. The control system incorporates pumps, digital pressure sensors, a microcontroller, solenoid valves and off-the-shelf components to deliver high and low air pressure in the desired temporal sequence to meter fluid flow and actuate microvalves. Importantly, our control system is portable, which is suitable for operating the microvalve-enabled microfluidic devices in the point-of-care setting.

## 1. Introduction

Automated microfluidic devices are amenable for carrying out complex multistep assays while minimizing sample volume, sample dilution and reagent use [1,2,3]. Microfluidic automation is frequently enabled by micromechanical valves (microvalves) also known colloquially as Quake-style valves [4]. Such microvalves may be used for routing and metering flow as well as for mixing [5]. While microfluidic devices with integrated microvalves have a small footprint (on the order of 1 cm^2^), they typically rely on bulky ancillary equipment (e.g., solenoid valves and pumps) to actuate the microvalves and flow of fluids. This makes it challenging to operate automated valve-enabled microfluidic devices at the point of use. Therefore, the objective of our study was to design a portable control system that may be deployed at the point-of-use setting to operate such devices.

Neonatology is one area where automated microfluidic devices may be particularly effective. Preterm newborns may have as little as 50 mL of total blood volume and are at an elevated risk for a number of health conditions including hypoglycemia [6], infections [7] and jaundice [8,9]. To improve health outcomes, preterm infants must be carefully monitored and screened for these conditions by measuring biomarkers in the blood. Although simple and accurate methods have been developed to measure blood glucose and bilirubin, traditional lab-based blood analysis requires 200–500 μL of blood per test. For critically ill infants who require continuous monitoring, blood draws may remove up to 50% of total blood volume during the first weeks of life, putting newborns at risk of anemia and infections [10,11,12,13]. Our team recently developed an automated valve-enabled microfluidic device that allows us to isolate plasma and then detect biomarkers based on an input of 5 μL of whole blood [14]. However, in this past study, we relied on external laboratory equipment and facilities (e.g., house air, solenoid valves, vacuum pump) to operate the microfluidic device. In the present study, we wanted to develop a portable control system to drive operation of the valve-enabled microfluidic device and detect an important biomarker of neonatal health from a small volume of human blood.

We note that there have been several previous reports of custom-made pneumatic control systems for actuation of microvalves [15,16,17]. In such reports, electronic and pneumatic components were engineered to create sophisticated control systems to operate automated microfluidic devices. However, some of these reports describe systems that are intended for laboratory use and for operation by experienced personnel [18,19]. Other reports have focused on miniaturizing pneumatic control systems for either pumping or valve actuation but not both [16,17,20]. Therefore, to the best of our knowledge, a control system capable of both microvalve actuation and fluid flow handling has not been reported to date. In addition, we are not aware of a report where a portable control system was used to drive operation of an automated microfluidic device that carries out a complex multi-step bioassay workflow.

**Figure 1:**
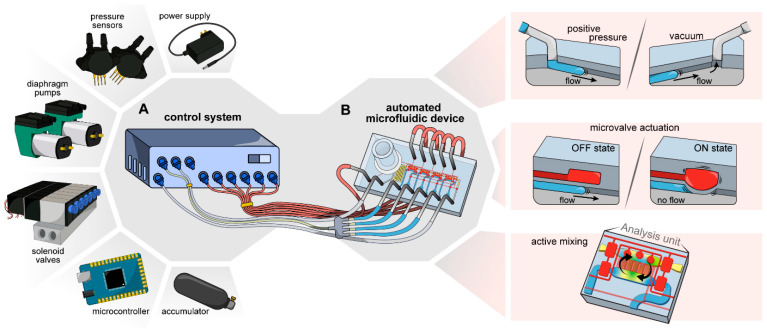
**A control system for automated operation of microfluidic devices.** (**A**) The control system comprises necessary pneumatic and electronic components to autonomously operate an automated microfluidic device. (**B**) Image of the microfluidic device and its functions. Negative pressure is applied to pull plasma into the device, while positive pressure is applied to flow solutions into the analysis unit and remove bubbles. Microvalves are first configured to connect analysis units in series for filling with plasma and are later reconfigured to sequester individual analysis units and commence mixing of plasma and glucose assay reagents. An analysis unit is comprised of plasma and reagent compartments (50 nL each).

In this paper, we sought to develop a compact control system for fully automated operation of a valve-enabled microfluidic device. This system and its components are described in Figure 1A. This control system was used to drive operation of a microfluidic device that performed a complex set of functions that included on-chip plasma separation, delivery of plasma into four analysis units and active mixing of plasma with assay reagents in these analysis units (Figure 1B). We demonstrated that a control system combined with this microfluidic device enabled automated blood processing and glucose detection in <15 min based on ~8 μL of whole blood.

## 2. Materials and Methods

### 2.1. Materials

Electronics and components used to build the control system are shown in the Bill of Materials as part of the Supplementary Information. For all experiments, materials used are listed below.

Polydimethylsiloxane (PDMS) base and curing agent kit (Sylgard 184) was purchased from Ellsworth Adhesives (Minneapolis, MN, USA). Tubing (Tygon^®^ ND-100-80 Microbore, 0.020″ ID × 0.060″ OD) was purchased from Cole Parmer (Vernon Hills, IL, USA). The plasma separation membrane (PSM) was obtained from Cobetter Filtration as a gift (Hangzhou, China). 6 mm glass cloning cylinders (Pyrex 31666), horseradish peroxidase (HRP), and the glucose assay kit (Am-plex™ red glucose/glucose oxidase assay kit) were purchased from Thermo Fisher Scientific (Waltham, MA, USA). Phosphate buffered saline (PBS) 1X was purchased from Corning (Corning, NY, USA). Chlorothrimethylsilane, 4-aminoantipyrine (4-AAP), glucose (D-(+)-Glucose), glucose oxidase (GOx), and the hemoglobin assay kit (MAK115) were purchased from MilliporeSigma (Burlington, MA, USA). The total bilirubin assay kit (BILT3) was purchased from Roche (Basel, Switzerland). Sodium salt of N-Ethyl-N-(2-hydroxy-3-sulfopropyl)-3-methoxyaniline (ADOS) was purchased from Dojindo (Kumamoto, Japan).

### 2.2. Design of the Control System

The objective of this manuscript is to show the fabrication of a control system to autonomously operate an automated microfluidic device for blood plasma analysis that has been previously reported [14]. To achieve this, we developed a control system that contains electronic and pneumatic components enclosed in a 280 × 280 × 80 mm acrylic box.

#### 2.2.1. Onboard Electronics

The onboard electronics of the control system consisted of a microcontroller (Arduino Mega 2560) connected to a custom printed circuit board (PCB), a diaphragm pump controller PCB (DFRobot), two diaphragm pumps, three digital pressure sensors (one for high and two for low pressure supply) and 13 three-way normally open solenoid valves. All onboard electronics were powered by a single 12 V power supply. 12 V were used to supply power to the diaphragm pumps, solenoid valves and high-pressure sensor. For the microcontroller and low-pressure sensors, voltage was stepped down with a 12 V to 5 V switching regulator (Recom Power) integrated into the custom PCB. The design of the PCB can be found in the following GitHub repository: https://github.com/RevzinLab/Control-System-for-Microfluidics (accessed on 30 October 2022).

#### 2.2.2. Pneumatic System

The pneumatics system is divided into high- and low-pressure systems for activation of microvalves and delivering sample/reagents, respectively. The high-pressure system comprised a diaphragm pump connected from the air outlet to a custom accumulator and sequentially to a 4-way splitter, connecting the accumulator to: (1) a digital high-pressure sensor, (2) a single solenoid valve used as a vent valve to release pressure, and (3) a 6-station manifold mounted with six solenoid valves operating as control valves for the microfluidic device. An air filter is connected to the air inlet of the diaphragm pump to avoid unwanted particles entering the system.

The low-pressure system enabled positive and negative pressure through two pneumatic circuits, one circuit connected to the air inlet of the diaphragm pump to obtain negative pressure, while the second one was connected to the air outlet of the diaphragm pump for positive pressure. For negative pressure, a 4-way splitter is used to interconnect the diaphragm pump air inlet, a solenoid valve used as a vent, a digital low-pressure sensor and a second solenoid valve that will be providing negative pressure to the microfluidic device. For positive pressure, the pneumatic circuit is similar to the high-pressure circuit, with the difference that a 4-station manifold with only three solenoid valves is used to operate fluid flow channels of the microfluidic device; the remaining station of the manifold is blocked to avoid leakage.

#### 2.2.3. Pressure and Solenoid Valves Control

To finely tune pressure in our system and control operation of solenoid valves, we created an algorithm using microcontroller software (Arduino IDE v1.8.19, Arduino, Somerville, MA, USA). We first calibrated our three digital pressure sensors against laboratory pressure regulators (LU-FEZ-N345, Fluigent, 67 Av. de Fontainebleau, 94270 Le Kremlin-Bicêtre, France) following the manufacturer’s recommendations to create reliable pressure readings. Then, we developed an algorithm with specific instructions to activate/deactivate diaphragm pumps and solenoid valves at specific time points based on the requirements of the assay. Diaphragm pumps and solenoid valves switching is achieved by activating/deactivating digital ports from the microcontroller. A commercial dual DC motor controller was used as an intermediate interface between the microcontroller and diaphragm pumps to deliver sufficient electrical current for activation. Similarly, we used a custom-made PCB with 16 Darlington transistor arrays (ULN2803A, STMicroelectronics, 39, Chemin du Champ des Filles, Geneva, Switzerland) to deliver current independently to each solenoid valve for activation. The Arduino code can be found in the project’s GitHub repository: https://github.com/RevzinLab/Control-System-for-Microfluidics (accessed on 30 October 2022).

### 2.3. Fabricating Microfluidic Devices and Connecting Them to the Control System

Fabrication of the microfluidic device was described in detail in our recent publication [14]. Briefly, two master molds were fabricated on silicon wafers by photolithographic patterning of SU-8 and AZ 10XT photoresists. These molds were replicated using polydimethylsiloxane (PDMS) to assemble a two-layer microfluidic device. This microfluidic device is comprised of a flow layer for sample and reagents and a control layer containing microvalves to handle fluid flow in the flow layer.

The flow layer was comprised of six inlets –plasma sample, air, and four for reagents- and five outlets -one for negative pressure and four for reagents-. The control layer contained six independent microvalves. The microfluidic device was coupled with the control system using medical grade tubing. The air inlet from the flow layer of the microfluidic device was connected to one of the solenoid valves from the positive pressure low-pressure circuit in the control system, while all four reagent inlets were connected to a second solenoid valve, which allowed pressurization of all four reagents inlets at the same time. The outlet of the sample chambers was connected to the negative pressure low-pressure system, this allowed for plasma separation from whole blood. On the other hand, each one of the microvalves from the control layer were connected to the six solenoid valves from the high-pressure circuit in the control system allowing independent activation/deactivation of each microvalve in the microfluidic device.

### 2.4. Design of Microfluidic Devices

The microfluidic device shown in Figure 1 integrated two modules: plasma separation and bioanalysis. The plasma separation module consisted of a plasma separation membrane (PSM) placed in the sample inlet and a collection microchannel that can hold plasma after separation from whole blood. After dispensing 8 μL of blood into the sample inlet, vacuum (−1 psig) was applied to the outlet of the plasma separation module to pull plasma into the collection microchannel. The PSM retained all blood cells allowing only plasma to enter the collection microchannels.

The bioanalysis module consisted of four analysis units for biomarker detection (see Figure 1B). Each analysis unit contained two 50 nL chambers for loading sample and glucose assay reagents. After the plasma was separated, it was pushed into the sample chambers at the same time that assay reagents were injected into the reagent chambers. Solutions in each analysis unit were efficiently homogenized by active mixing using two microvalves. This ensured that enzymatic reaction for glucose detection was not transport-limited and allowed for a signal to develop within 8 min of mixing. Enzymatic reactions were quantified by placing a microfluidic device on an inverted microscope with an integrated color camera (Primovert HDcam, Zeiss, Carl-Zeiss-Strasse 22, 73447 Oberkochen, Germany).

### 2.5. Operating a Microfluidic Device Using the Control System

To accomplish the desired workflow, our control system had to perform a set of specific functions. The workflow of two parts: (1) microvalve filling and activation, and (2) assay performance. Filling the microvalves consisted of loading microvalve tubing with DI water and connecting them to the microfluidic device and solenoid valves of the control system. After this, the control system was powered up and all microvalves were pressurized to 25 psig for 5 min to fill all microvalve channels with DI water. Afterwards, the algorithm entered a 10 min delay to allow preparation of the assay. During the 10 min delay, reagents were loaded into the tubing and connected to both the microfluidic device and control system. Tubing for the air inlet and vacuum outlet was connected as well. The microfluidic device was set on the inverted microscope and 8 μL of blood was dispensed into the sample inlet.

Performing glucose assay required six sequential steps. Schematics depicting the steps explained next are shown in Figure S1. (i) Microvalves connecting the PSM inlet with the vacuum outlet were deactivated to allow communication between the blood sample and negative pressure. (ii) Negative pressure (−1 psig) was applied for 30 s to the vacuum port to pull the plasma through the PSM into the collection microchannel. (iii) The PSM inlet microvalve was activated to block that inlet. Simultaneously, reagent and air inlet microvalves were deactivated and 1 psig was applied for 20 s to such inlets to fill reagent chambers and to push plasma from the collection microchannel into the sample chambers. (iv) A degassing step was performed to remove all air bubbles that could be trapped inside the reaction chambers. The outlet microvalve was activated and 4 psig were applied for 3 min to reagents and air inlets. (v) Reaction chambers were completely depressurized and all microvalves except for V3 (rounded microvalves on top of sample chambers) were activated. This sequestered each reaction chamber and prevented cross contamination between adjacent chambers. (vi) The next step consisted of mixing the contents of the sample and reagent chambers to develop the enzymatic reaction through active mixing. For this, we sequentially activated and deactivated microvalves V3 and V5 (microvalves separating sample and reagent chambers) for 8 min to actively mix solutions. At the end of this step, all microvalves were activated except for V3. At this point, image acquisition and quantification of enzymatic reaction began.

### 2.6. On-Chip Plasma Separation

We compared the quality of plasma isolated in a microfluidic device operated with the control system to a standard centrifugation method. For this, we used a microfluidic device that consisted only of the plasma separation module. The device had two inlets (plasma and air) and one outlet (vacuum). On top of the plasma inlet, a PSM (8 mm in diameter) was bonded and a sample of 8 µL of whole blood was dispensed on it. Empty tubing was connected to the air inlet and vacuum outlet. A simple algorithm consisting of two steps was used for plasma separation and collection: (1) −1 psig was applied to the vacuum outlet for 2 min to pull plasma into the collection microchannel. (2) 1 psig was applied to the air inlet to push plasma out of the device and into the outlet tubing for testing.

In parallel with on-chip blood processing, plasma was separated from the same blood sample using a centrifugation-based protocol. Briefly, blood was centrifuged at 1500× *g* for 12 min at 20 °C, blood cells formed a pellet at the bottom of the tube and plasma was aspirated out. Hemoglobin absorbance levels in both types of plasma samples were determined using a commercially available kit (MAK115, MilliporeSigma, 400 Summit Drive, Burlington, MA, USA) and a spectrophotometer (NanoDrop, Thermo Fisher Scientific, 168 Third Avenue, Waltham, MA, USA).

### 2.7. On-Chip Enzymatic Assay for Glucose Detection

An enzymatic colorimetric assay for glucose detection was described by us previously [14,21]. Briefly, the assay reagents were reconstituted at the following concentrations in order to increase contrast for absorbance-based measurement in the device: GOx at 70 U/mL, HRP at 117 U/mL, ADOS at 3.6 mM and 4-AAP at 3.1 mM. Glucose solutions of varying concentrations (from 0 to 10 mM) were prepared and infused into a microfluidic device to create a 1:1 sample to reagent mixture. Upon mixing of the glucose solutions and the reagents, the solution developed a magenta-colored product with the absorbance intensity correlating to glucose concentration.

After characterizing the performance of the device using known glucose concentrations, we assessed glucose in blood. Blood samples were acquired from the Mayo Clinic blood bank and were collected into 6 mL EDTA-coated tubes. Because these were de-identified leftover blood bank samples, no IRB protocol was required. Five blood samples were used to assess the performance of the control system and automated microfluidic devices. For each sample, three analysis units were used to account for on-chip variability. Two-point calibration was run in the same device by flushing the analysis units with 1X PBS to establish a 0 mM value and then with an 8 mM glucose solution. Images were acquired for sample and calibration solutions to analyze and determine glucose concentration in the samples.

### 2.8. Image Acquisition and Analysis

Colorimetric enzymatic reaction in microfluidic devices was assessed by acquiring images in the brightfield channel using an inverted microscope and a color camera. Image analysis and data plotting was performed automatically using a custom-made MATLAB (2021b, Mathworks) script. For this, images were trimmed, and the intensity of a rectangle of 300 × 100 px in the middle of the reagent chamber was measured for each color channel in the image (R, G, and B). Data was converted into the CMYK color space, and magenta channel values were used to determine the glucose concentrations of the samples. For the calibration curve, magenta intensity from images was plotted as a function of the corresponding concentration of glucose in the solution. For whole blood samples, the glucose concentration of plasma was determined by comparing against the 0- and 8-mM solutions. The MATLAB script can be found in the project’s GitHub repository: https://github.com/RevzinLab/Control-System-for-Microfluidics (accessed on 30 October 2022).

## 3. Results and Discussion

The goal of this study was to develop a control system that could be used to drive operation of a valve-enabled microfluidic device performing blood processing and biomarker detection. This microfluidic device, reported by us recently [14], allowed us to isolate plasma from a small volume of blood and then carry out mix-and-read assays. The device contains a plasma separation module upstream of the bioanalysis module where the sample is mixed with reagents to carry out an assay and detect a biomarker of interest. Operation of this microfluidic device typically involves equipment or facilities common in a research laboratory, but uncommon in the point-of-care setting: (1) a source of high pressure (>20 psig, air compressor, in house pressure source, etc.) is needed to actuate Quake-style valves in microfluidic devices. The source of high pressure is connected to external solenoid valves that in turn are connected to a microcontroller operated by a graphic user interface (GUI). The GUI allows the user to control each microvalve or actuate microvalves in a prescribed sequence. (2) A source of low pressure (0–5 psig) to push the sample through the device at the desired flow rate. In this paper, we wanted to incorporate these capabilities into a portable system.

### 3.1. Design and Assembly of the Control System

**Figure 2:**
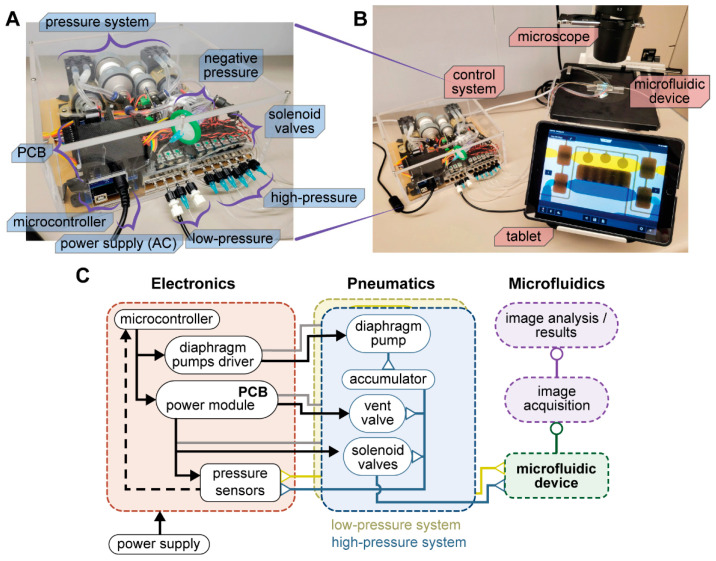
**Automated control system for microfluidic devices.** (**A**) Photograph of the control system describing the main components. (**B**) Setup of the microfluidic device on an inverted microscope while operated by the control system. (**C**) Diagram of component connections inside the control system and its interface with the microfluidic device.

To create a portable system, we sought to eliminate the need for an external pressure source and regulation. We designed a control system that is compact (280 × 280 × 80 mm) and only requires connection to alternating current (AC) to function, shown in Figure 2A,B. The system comprises a microcontroller and electronics that efficiently control pneumatic elements to deliver precise pressure and, at the same time, activate/deactivate all six microvalves in the microfluidic device. The pneumatic system was divided into two, a high-pressure subsystem to control all microvalves, and a low-pressure subsystem to inject solutions into the device.

We used two miniature diaphragm pumps (mini-pumps), one for each pressure subsystem. Each mini-pump was connected to an accumulator to build up pressure and the accumulator to three different components via a 4-way splitter: a digital pressure sensor, a vent valve to release excess pressure, and a manifold with solenoid valves. The manifold for the high-pressure subsystem interfaced six 3-way solenoid valves to six microvalves in the microfluidic device. Each solenoid valve would then supply the on-chip microvalve with pressurized air (>20 psig) or with atmospheric pressure to achieve an activated or deactivated microvalve state, respectively. The low-pressure subsystem interfaced only three solenoid valves to the device with pressure in the range of −5 to 5 psig. A diagram of all components in the control system is shown in Figure 2C. After all electronic and pneumatic components were obtained, we assembled the system and put it inside an acrylic box. The assembly of the system took ~4 h to complete.

### 3.2. Onboard Pressure Generation and Regulation

**Figure 3:**
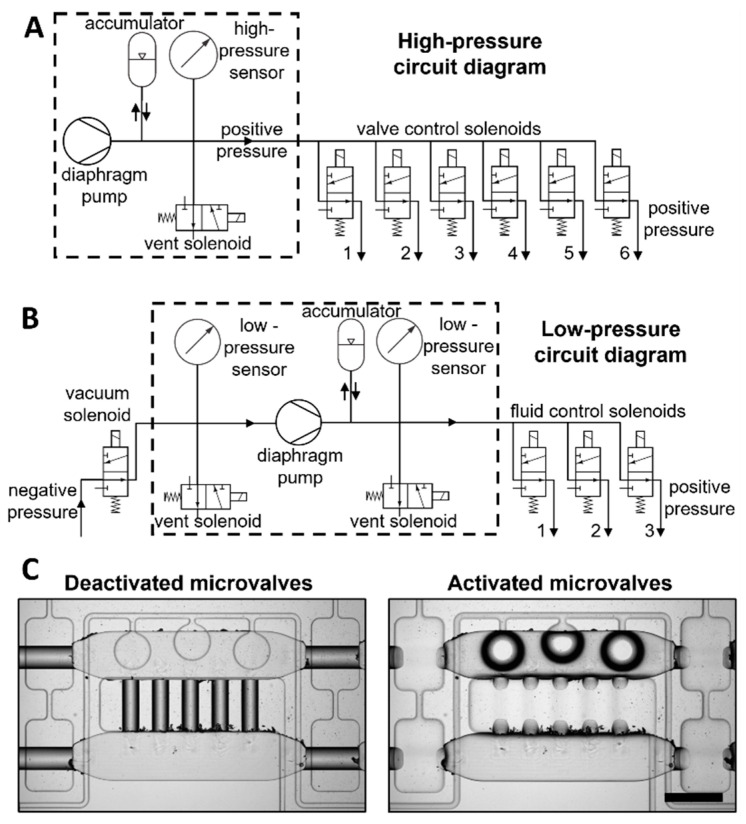
**Diagrams describing high- and low-pressure circuits of the control system.** (**A**) Diagram for the high-pressure circuit of the control system showing all components used to generate positive pressure to actuate all microvalves. The outlets 1 to 6 from the solenoid valves are connected to the microvalves in the microfluidic device. (**B**) Diagram for the low-pressure circuit to generate both positive and negative pressure to control the flow layer of the microfluidic device. The range of pressure the system provides is from −5 to 5 psig. The outlets 1 to 3 from the solenoid valves are connected to the fluid flow channels from the microfluidic device. (**C**) Microvalves actuation using the control system. Scale bar = 500 µm.

We used a microcontroller board and off-the-shelf electronics to control the pneumatic components described above. One dual DC motor controller interfaced both mini-pumps with the microcontroller, allowing for their individual activation. Pressure regulation was achieved by setting the mini-pump, digital pressure sensor, and the vent valve in a feedback loop such that the mini-pump increased the pressure in the accumulator to a setpoint while the vent solenoid released the excess pressure from the accumulator to the atmosphere. The pumping and venting process was monitored by the pressure sensor connected in parallel with the accumulator and was controlled with an in-house control algorithm. In this manner, the high-pressure system could deliver up to 25 psig with 1% precision (Figure S2A) to activate the microvalves. The pneumatic connections of the high-pressure system are shown in Figure 3A. The digital pressure sensor and solenoid valves were interfaced to the microcontroller via a custom-made PCB (Figure S3).

The low-pressure system was configured in a similar fashion but contained an additional pressure regulation component for negative pressure. By connecting a negative pressure regulator to the inlet of the mini-vacuum pump and a positive pressure regulator to the outlet of the mini-vacuum pump, negative and positive pressure sources could be achieved with a single system, as shown in Figure 3B. Negative pressure was generated by the mini-pump and regulated in the same manner as the high- and low-pressure subsystems, with the difference that there was not an accumulator connected. The low-pressure system could deliver pressure in the range of −5 to +5 psig within 1% precision and with 3% precision at 0.5 psig (Figure S2B,C).

### 3.3. Automated Isolation of Plasma from Blood Using a Microfluidic Device

**Figure 4:**
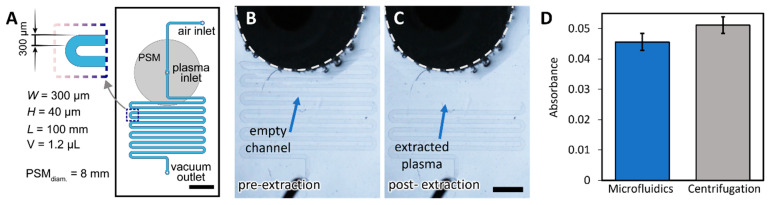
**Plasma separation quality assessment.** (**A**) Schematic of the microfluidic device used for plasma separation (modified from ref. [14]). Scale bar = 3 mm. The gray circle denotes the plasma separation membrane (PSM), and the blue channels the plasma collection microchannel. (**B**) Microfluidic device showing the PSM (dark area with dotted line) where the sample is deposited, and an empty collection microchannel before plasma extraction. (**C**) During plasma extraction, plasma travels through the PSM filling the collection microchannel, while all blood cells are retained in the PSM. Scale bar = 2 mm. (**D**) Absorbance values for plasma separated using the microfluidic device and control system compared to separation by centrifugation.

As an initial experiment, we tested the capabilities of our control system to separate plasma from whole blood in a simple microfluidic device. The microfluidic device consisted of a single serpentine microchannel capable of retaining up to 1 μL of plasma. A plasma separation membrane (PSM) was bonded to an inlet of the device and used to deposit the whole blood sample (8 μL). The schematic of the device is shown in Figure 4A. Upon application of negative pressure (−1 psig) to the outlet of the device, all blood cells were retained in the PSM and plasma entered the serpentine, filling it completely, as shown in Figure 4B,C. The extracted plasma was collected and kept in a collection tube. This was repeated three times to obtain three samples of plasma from the same blood sample from three different devices. The remaining blood was used to separate plasma by centrifugation, the gold standard method. Hemoglobin levels were detected in all samples using a commercial kit and a spectrophotometer and compared directly between both separation methods. As seen in Figure 4D, hemoglobin levels were similar in a microfluidic device compared to a standard centrifugation-based method. This result demonstrated that the control system could be used to drive automated separation of plasma from blood.

### 3.4. Automated Operation of a Microfluidic Device Performing Enzymatic Glucose Assay

**Figure 5:**
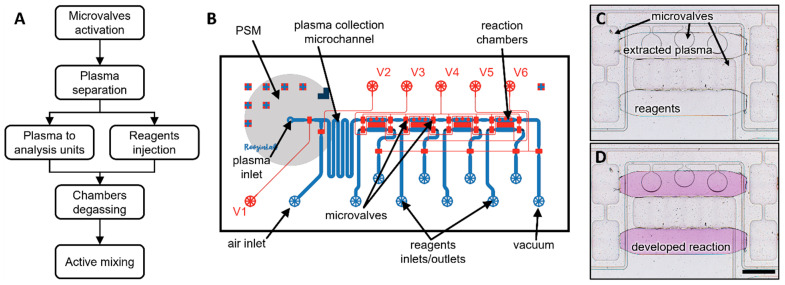
**Using control system to perform a mix-and-read assay in a microfluidic device.** (**A**) A sequence of steps performed by the control system and a microfluidic device. (**B**) Schematic of the automated microfluidic device for plasma separation and biomarkers analysis. The blue channels represent fluid flow channels, while the red channels represent all microvalves. (**C**) Micrograph showing the plasma and reagent compartments before mixing. Enzymatic reaction has not yet started. (**D**) Micrograph of the same analysis unit shown after 8 min of actively mixing contents of plasma and reagent compartments. Intensity of magenta color correlates with glucose concentration in the sample. Scale bar = 500 µm.

Having validated automated separation of plasma, we proceeded to employ the control system for automated performance of an enzymatic glucose assay. A systems-level flow diagram of the functions performed in the microfluidic device is shown in Figure 5A. Before starting, the microfluidic device is set on the stage of an inverted tabletop microscope that has an integrated color camera. An iPad was used to acquire images. All microvalves were connected to the solenoid valves with tubing filled with DI water. The algorithm started by activating all six microvalves and pressurizing the accumulator to 25 psig to fill all microvalve channels. Immediately afterwards, all reagent tubing was loaded and connected to the device. Empty tubing was connected to the air inlet and vacuum outlet. A whole blood sample of 8 µL was then dispensed into the PSM. Following this, the control system performed six sequential steps that relied on the activation/deactivation of specific microvalves and pressurizing inlets/outlets to create fluid flow and mix sample with reagents. Figure 5B shows the schematic of the microfluidic device depicting fluid flow channels and microvalves.

During the assay, the control system performed the next steps automatically: after the blood sample was deposited on the PSM, plasma was pulled into the collection microchannel by applying negative pressure to the outlet. Once collected, plasma was moved into the sample chambers, and reagents were loaded into the reagent chambers in parallel. At this point, the valves separating sample and reagent microchambers were actuated to prevent mixing. A degassing step was performed to remove air trapped inside the reaction chambers by pressurizing sample and reagents chambers for 3 min to allow air to diffuse-out through the PDMS. This step was crucial, as bubbles residing inside the chambers could prevent efficient mixing of solutions. Each bioanalysis unit was comprised of a sample microchamber and a reagent microchamber. One unit was sequestered from the neighboring unit by actuation of valves 4 and 5 and then the contents of the two microchambers within each unit were mixed to commence enzymatic reaction.

Active mixing is a key component of our assay. Microfluidic devices often rely on diffusion for mixing of solutions, which requires waiting times of tens of minutes to hours, depending on the dimensions of the microchambers and solutions being mixed. To accelerate mixing, we sequentially activated two microvalves (V3 and V5) to create chaotic flow that moves solutions back and forth between the sample and reagent chambers. Such active mixing efficiently homogenizes the solutions and allows for the enzymatic glucose reaction to be completed in <8 min [14]. Using the control system, we mixed solutions for 8 min during the active mixing step. Images were acquired from three chambers in the device and analyzed to determine glucose concentration. Figure 5C,D shows one bioanalysis unit before and after mixing of glucose with assay reagents. As may be seen from Figure 5D, mixing of the glucose sample (8 mM) with assay reagents produces an intense magenta color.

### 3.5. Image Analysis to Quantify Colorimetric Enzymatic Glucose Assay

**Figure 6:**
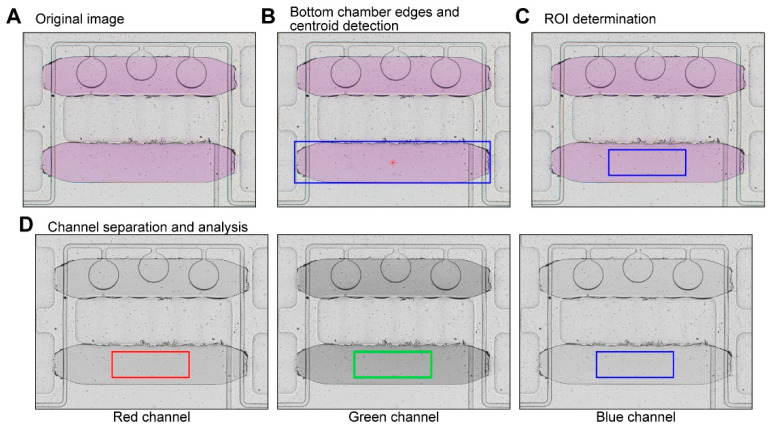
**Automated image analysis.** We used a MATLAB script to automatically analyze acquired images and generate concentration values. (**A**) Original image acquired using an inverted microscope. (**B**) The reagents chamber was detected, and its edges (blue box) and geometrical centroid (red star) were determined for analysis. (**C**) A ROI of 100 × 300 px (blue box) was created for analysis. (**D**) Each color channel was separated and analyzed individually for the posterior calculation of magenta intensity in the ROI (color boxes in each image).

We developed a custom image analysis script using MATLAB to analyze the colorimetric reaction autonomously upon completing the fluid manipulation step. The core function of the program is to calculate the intensity of the images acquired at the end of the enzymatic reaction. The software first opened all acquired images and aligned them to match the position of the bottom chambers in all of them. Then, the sample image was opened (Figure 6A) and binarized to locate the sample and reagent chambers. The sample chamber was discarded, and the edges and geometrical centroid of the reagents chamber was determined (Figure 6B). We did not use the sample chamber for analysis as the edges of the rounded microvalves could interfere with the analysis. A region of interest (ROI) in the middle of the reagents chamber (100 × 300 px) was determined for analysis. Then, each color channel (red—R, green—G, blue—B) from the original image was separated and the average intensity of the ROI was calculated for each channel (Figure 6D). The pixel intensity values in all color channels were converted to the CMYK color space to determine magenta intensity. The same process was repeated for each image in all experiments.

### 3.6. Detecting Glucose in Blood Using a Microfluidic Device Operated by the Control System

**Figure 7:**
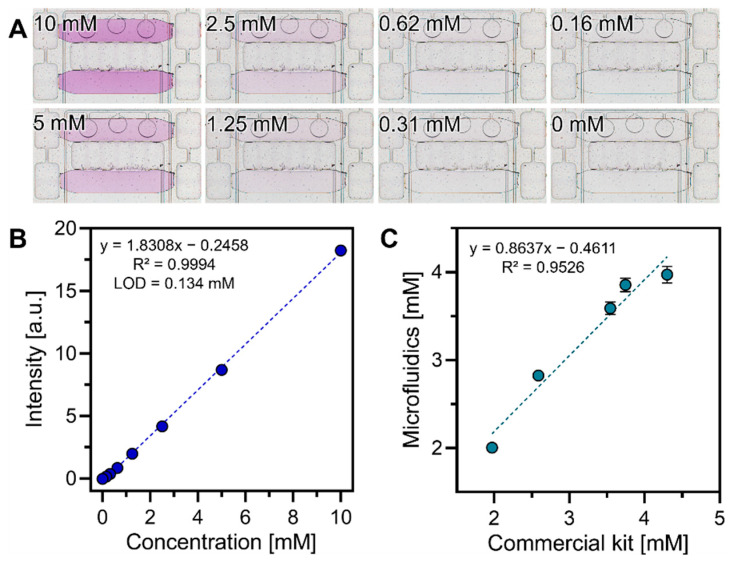
**Performing glucose detection assays in a microfluidic device operated by the control system.** (**A**) Images of microchambers with different concentrations of glucose. Scale bar = 500 µm. (**B**) Correlating intensity of magenta color and glucose concentration to construct a calibration curve. Limit of detection for this assay was 0.134 mM (*n* = 3). (**C**) Comparing levels of glucose in blood determined using a microfluidic device and a standard glucose kit (*n* = 3). 95% confidence intervals are plotted as grey dotted lines.

We first used solutions with known glucose concentrations to create a calibration curve. For this set of experiments, glucose solutions were introduced by connecting tubing to the plasma inlet and drawing the solutions into the sample chambers. All other steps were performed as described above. The reactions were developed by mixing of glucose solutions ranging from 0 to 10 mM with assay reagents (Figure 7A). Images from three chambers were acquired and analyzed using the MATLAB script and intensity values plotted as a function of glucose concentration as shown in Figure 7B.

These results demonstrate that a microfluidic device operated by the control system provides a limit of detection (LOD) of 0.134 mM glucose. This is comparable to limits of detection reported by us previously for an automated microfluidic device controlled by standard laboratory equipment [14].

As the final step in assessing the utility of the microfluidic device operated by the control system, we wanted to benchmark glucose detection from whole blood in our system against a commercial glucose assay kit. We obtained five blood samples from the Mayo Clinic blood bank and then split each sample in two parts, one was analyzed in a microfluidic device, another using a commercial kit. We note that glucose levels in a microfluidic device were established based on a two-point calibration using 0- and 8-mM glucose. Figure 7C compares glucose detection results from a microfluidic device and a commercial kit and shows excellent agreement between the two approaches (r^2^ = 0.96). We note, however, that a microfluidic device operated by the control system allowed us to fully automate blood processing and glucose detection steps, providing a result within 30 min based on an input of a few microliters of blood. Conversely, the standard assay was carried out using 0.5 mL of blood, required manual handling steps and centrifugation, and was completed in ~2 h. The values for the data plotted in Figure 7B,C can be found in Tables S1 and S2, respectively.

## 4. Conclusions

In this work, we described the development of a control system that drives a valve-enabled microfluidic device to perform a sophisticated workflow of plasma isolation from whole blood followed by detection of a model biomarker (glucose) using an enzymatic assay. The control system was portable (280 × 280 × 80 mm) and incorporated components to apply and regulate pressure for actuating microvalves and metering flow. This control system eliminated the need for external pumps and/or source of pressure typically used for operating Quake-style valves in microfluidic devices. Microfluidic devices operated by the control system were shown to efficiently isolate plasma from microliter volumes of whole blood. Furthermore, an integrated automated workflow of plasma separation and glucose detection performed in the microfluidic device yielded similar results (glucose concentration) compared to a standard laboratory-based workflow involving manual handling steps, including centrifugation-based plasma separation. Our study addresses an important but often overlooked question of how to operate sophisticated microfluidic devices with Quake-style valves at the point-of-care.

We note that the focus of this study was on controlling operation of a microfluidic device and that a signal transduction strategy was not miniaturized. There are multiple reports describing miniature optical transducers (either microscope objectives or cell phone attachments) [22,23,24] that may be integrated with our control system to enable sample-in/answer-out functionality and fully autonomous operation at the point-of-care. Implementing a signal transducer with the control system and demonstrating detection of multiple analytes in the same automated device represent future directions for this project.

## Data Availability

Not applicable.

## Supplementary Materials

The following are available online at https://www.mdpi.com/article/10.3390/bios12121160/s1, Figure S1: Glucose assay steps in the microfluidic device, Figure S2: Pressure systems stability as a function of time, Figure S3: Onboard PCB Schematic, Table S1: Calibration curve data, Table S2: Blood samples data, Bill of Materials.
