# Supplementary material for: A Compact Control System to Enable Automated Operation of Microfluidic Bioanalytical Assays
Source: Biosensors (Basel). 2022 Dec 13;12(12):1160. doi: 10.3390/bios12121160 (PMC9775492; doi:10.3390/bios12121160)
Supplement: Supplementary file 1 [file biosensors-12-01160-s001.zip › biosensors-1931779-supplementary.pdf]

Supporting Information for

A Compact Control System to Enable Automated Operation of Microfluidic Bioanalytical Assays

Alan M. Gonzalez-Suarez[1]*, Alexander Long[1,2], XuHai Huang[1] and Alexander Revzin[1]*

[1]*Department of Physiology and Biomedical Engineering, Mayo Clinic, Rochester, MN 55901, USA*

[2]*Department of Biology, St. Olaf College, Northfield, MN, 55057, USA*

*Corresponding authors: GonzalezSuarez.Alan@mayo.edu, Revzin.Alexander@mayo.edu

CONTENTS

Supplementary Figures

Figure S1. Glucose assay steps in the microfluidic device ········ **S3**

Figure S2. Pressure systems stability as a function of time ········ **S4**

Figure S3. Onboard PCB Schematic ········ **S5**

Supplementary Tables

Table S1. Calibration curve data ········ **S6**

Table S2. Blood samples data ········ **S6**

Bill of Materials

1. Components of the microfluidics control system ········ **S7**

2. PCB components for the microfluidics control system (main PCB) ········ **S8**

3. Pressure regulator components ········ **S9**

4. Tubing and fittings (non-exhaustive) ········ **S11**

SUPPLEMENTARY FIGURES

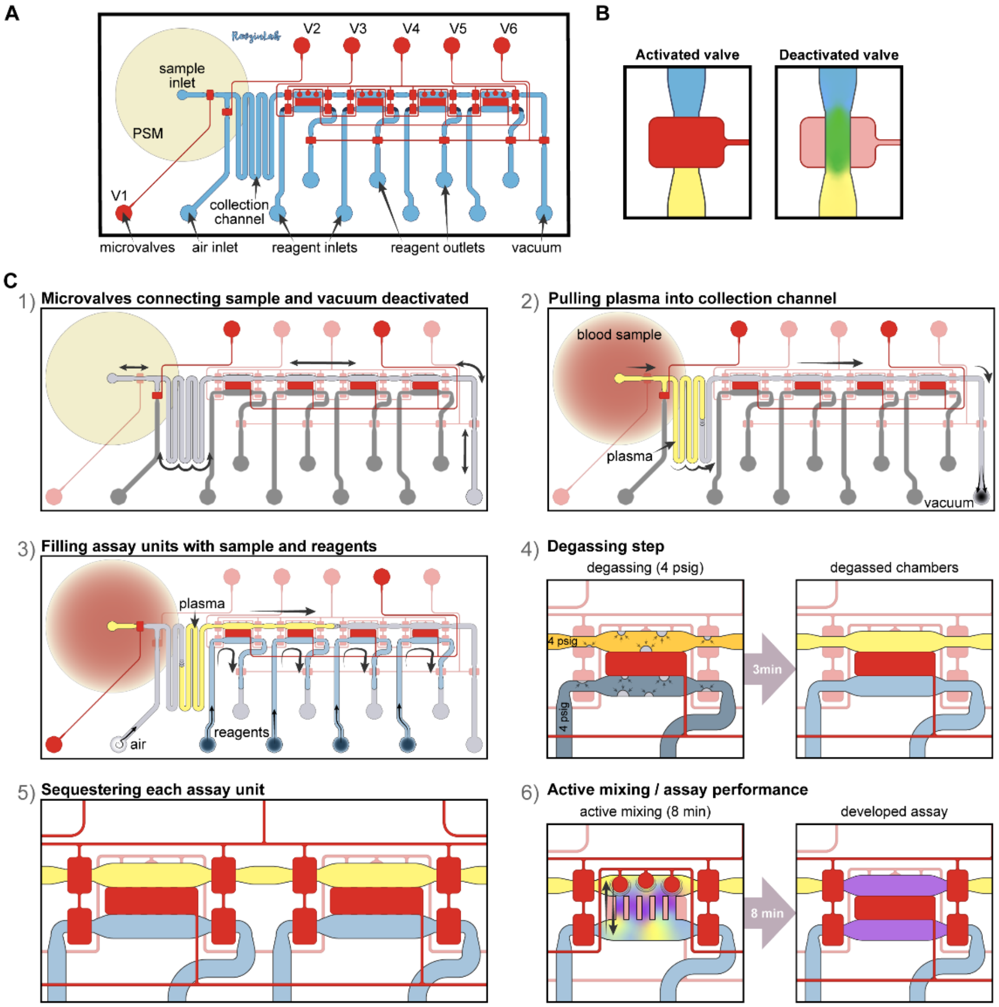

Figure S1. Glucose assay steps in the microfluidic device. A) Schematic of the microfluidic device with incorporated microvalves and the PSM. **B)** Activated and deactivated states of the microvalves for schematics shown in C). **C)** Steps performed in the microfluidic device by the control box to develop a glucose assay in a blood sample. This protocol starts with all microvalves activated. *(1)* First, the microvalves V1, V3, V4 and V6 are deactivated to allow communication from the sample inlet to the vacuum port. *(2)* The blood sample is deposited in the PSM and -1 psig applied to the vacuum port to pull the plasma into the collection channel. *(3)* The microvalve V1 is activated and V2 deactivated. Following this, 1 psig are applied to the air and reagents inlets to push the plasma and reagents into the sample and reagents chambers, respectively. (4) The microvalve V6 is activated, closing all outlets, and 4 psig are applied ot the air and reagents inlets. This step removes any air bubbles trapped inside the assay chambers. (5) The microvalves V2 and V4 are activated to sequester each assay unit independently and prevent cross contamination between adjacent chambers. (6) The contents of the sample and reagents chambers are actively mixed for 8 min to develop the glucose enzymatic reaction. For this, the microvalves V3 and V5 are activated and deactivated sequentially every 100 ms, resulting in a fully developed reaction after the 8 minutes. After this, images of all assay units are acquired.

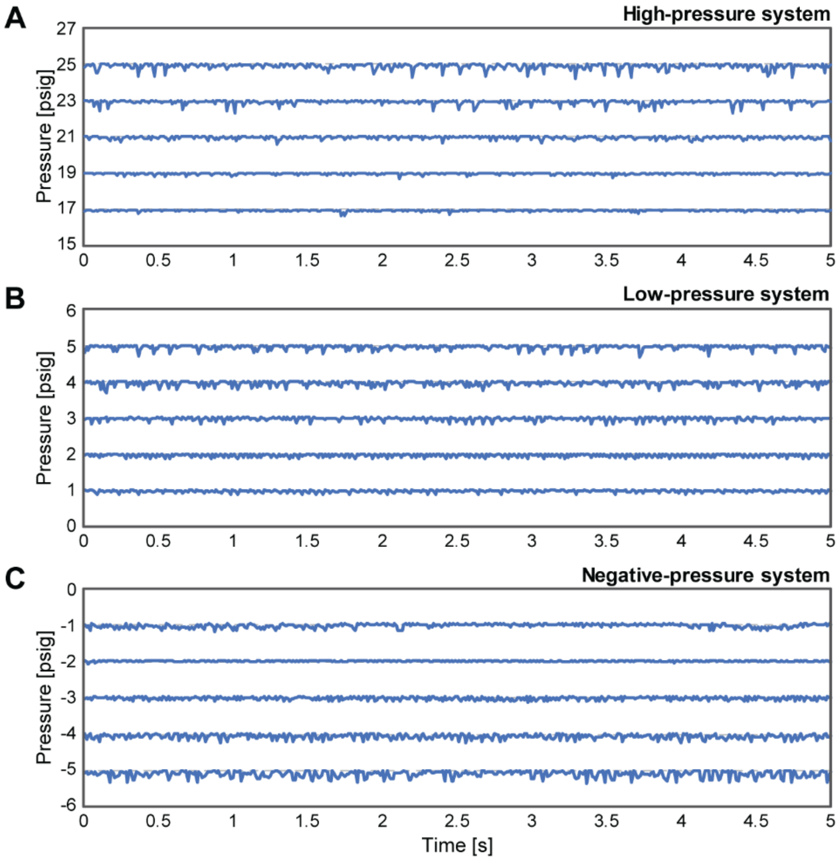

Figure S2. Pressure systems stability as a function of time. A) Graph showing the stability of the high-pressure system for up to 25 psig. **B)** Stability of the low-pressure system from 0 to a maximum pressure of 5 psig. **C)** Stability of the second branch of the low-pressure system that provides negative pressure ranging from 0 to -5 psig.

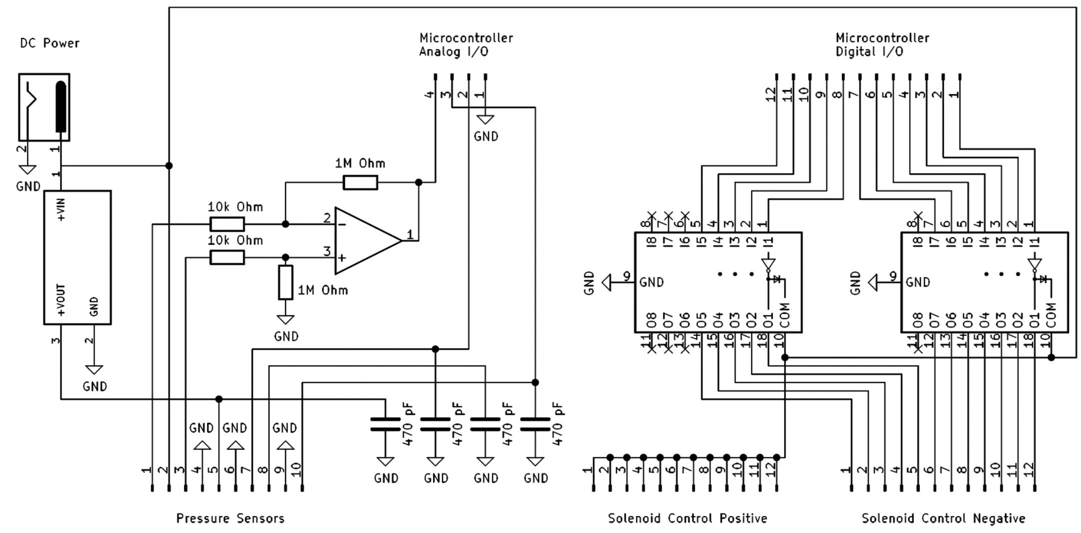

Figure S3. Onboard PCB schematic. Electrical schematic of the onboard PCB for controlling the solenoid valves and pressure sources. The PCB shows connections from the microcontroller to two amplifiers to power up the solenoid valves, and connections to receive the readings coming from the pressure sensors. The system is powered up with an external 12V power source.

SUPPLEMENTARY TABLES

Table S1. Calibration curve data. Information obtained from the glucose calibration curve experiment shown in **Figure 7B**, showing the average magenta color intensity from the microfluidic chambers. The standard deviation was calculated from three technical replicates (n = 3).

Concentration [mM]	Mean Intensity [mM]	Std. dev.
**10**	18.226	0.244
**5**	8.684	0.065
**2.5**	4.158	0.011
**1.25**	1.985	0.012
**0.625**	0.842	0.010
**0.313**	0.354	0.004
**0.156**	0.149	0.002
**0**	-0.033	0.003

Table S2. Blood samples data. Values obtained from glucose analysis in blood when comparing our microfluidic system against a commercial kit. Five blood samples were analyzed, and the data plotted is shown in **Figure 7C**. The standard deviation was obtained by analyzing three technical replicates (n = 3).

Sample	Commercial kit [mM]	Microfluidics [mM]	Std. dev.
**1**	3.744	3.856	0.077
**2**	1.969	2.004	0.053
**3**	2.585	2.825	0.049
**4**	4.300	3.972	0.095
**5**	3.543	3.588	0.072

Components of the microfluidics control system

This document lists all components needed to build a microfluidics control system. It is organized in several sheets. This sheet lists the components for the overall system; the BOMs for the main PCB and the pressure regulator are listed in their own sheets, as is a list of necessary fittings and tubes to connect all components.

Description	Supplier	Supplier part number	Quantity	Unit price (USD)	Extended price (USD)	Notes
Printed circuit board, assembled			1	70.87	70.87	
Pump, 12V DC, 32 psi	Amazon	B0192KPBCK	1	21.65	21.65	Alternatively: Parker #H085-11 pump ($99) + Parker 00329-10-A45S mount ($14). 2 pumps can also be used, in case a vacuum supply is needed.
Moisture filter	McMaster-Carr	41575K61	1	13.74	13.74	Place after pump to prevent build up of water in tubes
Pressure regulators			2	200.825	401.65	Alternatively, Parker pressure controllers (e.g. 990-005103-015) can be used; however, they will need a larger pressure supply than the on-board pump
Solenoid valves	Pneumadyne	S10MM-31-12-2, S10MM-30-12-2	32	27.49	879.68	Part numbers refer to normally-open and normally-closed valves, respectively. Either can be used, depending on application. But you cannot have both NO and NC valves on the same manifold.
Manifold for valves, 8 stations	Pneumadyne	MSV10-8	4	25.41	101.64	
Check valve	Pneumadyne	C050401	1	5.68	5.68	Place after pump, to prevent large oscillations in pressure when pump is running
10-32 UNF plug (25-pack)	Pneumadyne	SPG-10	0	13.25	0	Plugs for unused manifold stations. Not necessary if getting equal number of valves and manifold stations.
Cover plates for manifolds	Pneumadyne	MSV10-CP	0	5.06	0	
Tubing, misc. fittings	McMaster-Carr	Various			169.86	See details on 'Tubing and fittings' sheet.
M3 screws, standoffs	Amazon	B06XQ33Y9X	1	11.99	11.99	Provided as example; M3 standoffs are useful for mounting the PCBs and some other components; all holes are M3 threaded or clearance.
M3 anti-vibration screws	Amazon	B071WBZC9K	1	7.88	7.88	Useful when mounting the pump, to absorb vibration
AC -> 12V DC adapter	Amazon	B015G8E7JU	1	16.99	16.99	12V, 5A minimum supply
Enclosure	eBay	122068688371	1	26.27	26.27	Cheval Group, Model #: 2U-D1-W2. http://www.chevalgrp.com/standalone.php ; alternatively, build custom enclosure
				**Total:**	**1727.9**	

PCB components for the microfluidics control system (main PCB)

Also includes connectors for components that plug into the PCB

Description	Ref.	Supplier	Supplier part number	Quantity	Unit price (USD)	Extended price (USD)	Notes
PCB		DirtyPCBs.com	n/a	1	29	29	Unit price listed is for 10 boards. Extended price reflects this.
ESP32 dev kit C	E1	Amazon	B01N0SB08Q	1	12.95	12.95	
Step-up converter, 24V out		Amazon	B06XWSV89D	0	10.99	0	Includes 5 units. Only necessary if using 24V pressure regulators (Parker OEM / VSO series)
IO expander (PCA9698)	U6	Digikey	568-3241-5-ND	1	4.53	4.53	
Darlington arrays (ULN2803A)	U1, U2, U3, U4, U5	Digikey	296-15777-1-ND	5	1.53	7.65	
Quad Op-Amp (TLV2374)	U8	Digikey	296-11967-1-ND	1	2.01	2.01	
DC-DC converter, 5V out (Recom R-78E5.0-1.0)	U7	Digikey	945-2201-ND	1	3.26	3.26	
**Connectors**							
Barrel jack	J1	Digikey	CP-063AH-ND	1	1.36	1.36	
5x2 header	J3, J4, J5, J8	Digikey	ED1543-ND	4	0.29	1.16	
2x1 header, locking	J6,J7	Digikey	A1921-ND	2	0.12	0.24	
2x1 receptacle		Digikey	A31017-ND	2	0.18	0.36	Connector for pump
4x1 header, locking	J9,J10,J11	Digikey	A19431-ND	3	0.19	0.57	
4x1 receptacle		Digikey	A31004-ND	3	0.36	1.08	Connector for Parker pressure regulators
5x2 connector set		Amazon	B00W8U8CSC	1	4.99	4.99	Connector for solenoid valves
**Resistors**							
1k6, 0603	R1, R2	Digikey	P1.60KHCT-ND	3	0.1	0.3	Part numbers can of course change; any 0603 resistors with 1% tolerance can be used
1k8, 0603	R5, R6, R7, R9	Digikey	MCT0603-1.80K-CFCT-ND	6	0.152	0.912	
2k7, 0603	R3, R4	Digikey	RHM2.70KADCT-ND	3	0.14	0.42	
3k3, 0603	R8, R10	Digikey	311-3.30KHRDKR-ND	3	0.026	0.078	
				**Total**		**70.87**	

Pressure regulator components

Number of pressure regulators	2

Component / value	Ref.	Supplier	Supplier part #	Qty per board	Total qty	Unit price (USD)	Extended price (USD)	Notes
PCB		DirtyPCBs.com		1	2	11.95	11.95	Unit price listed is for 10 boards. Extended price reflects this.
**Connectors**								
4x2 female angled	J3	Digikey	S5518-ND	1	2	0.96	1.92	
2x1 female angled	J4	Digikey	S5438-ND	1	2	0.43	0.86	
USB micro B	J1	Mouser	798-ZX62D-B-5PA830	1	2	0.7	1.4	
**Diodes**								
Diodes, SOD-323	D1,D2,D3, [	Mouser	621-1N4148WS-F	3	6	0.156	0.936	
Green LED, 0603	D4	Mouser	604-APHD1608LCGCK	1	2	0.46	0.92	
Schottky diodes, SOD-323	D6, D7	Mouser	863-NSR0530HT1G	2	4	0.142	0.568	
**IC**								
Atmega32u4	U3	Mouser	556-ATMEGA32U4-AU	1	2	4.8	9.6	
MOSFET, Dual N-channel	Q1	Mouser	621-DMN6070SSD-13	1	2	0.53	1.06	
**Misc**								
Polyfuse	F1	Digikey	F2902CT-ND	1	2	1.06	2.12	
Switch, B3U-1000P	SW1	Mouser	653-B3U-1000P	1	2	0.97	1.94	
Crystal, 8MHz	Y1	Mouser	520-8.0-18-23G-JGNT	1	2	0.73	1.46	
**Voltage regulators**								
3.3V out	U1	Mouser	595-TLV76033DBZT	1	2	0.41	0.82	
5V out	U2	Mouser	595-TLV76050DBZT	1	2	0.41	0.82	
**Capacitors**								
0.1uF, 0603	C1	Digikey	1276-1013-1-ND	1	2	0.1	0.2	
1uF, 0603	C2,C7 ,C9	Digikey	1276-1946-1-ND	3	6	0.1	0.6	
10uF, 0805	C3	Digikey	1276-6456-1-ND	1	2	0.15	0.3	
18pF, 0603	C4,C5	Digikey	399-7866-1-ND	2	4	0.12	0.48	
**Resistors**								
22, 0603	R1,R2	Digikey	1276-4511-1-ND	2	4	0.033	0.132	Part numbers can of course change; any 0603 resistors with 1% tolerance can be used
10k, 0603	R3	Digikey	RHM10KADCT-ND	1	2	0.14	0.28	
3k2, 0603	R4	Digikey	311-3.24KHRCT-ND	1	2	0.1	0.2	
1k8, 0603	R5	Digikey	MCT0603-1.80K-CFCT-ND	1	2	0.152	0.304	
2k7, 0603	R6	Digikey	RHM2.70KADCT-ND	1	2	0.14	0.28	
680, 0603	R7	Digikey	P680HCT-ND	1	2	0.1	0.2	

Pressure sensor	U4/U5	Mouser	785-HSCDRRT030PGAA5	1	2	37.15	74.3	Various options available. Any Honeywell HSC or SSC series sensor with an analog or SPI interface and 8-pin DIP package will work. Supply voltage can be 3.3V or 5V.
**Non-PCB components**								
Parker VSO LowPro		Parker	935-300120-000	2	4	70	280	12V valves are recommended, but 5V and 24V will also work.
Manifold, machined 6061 aluminum				1	2	2	4	Price can vary a lot between making the manifold oneself (~2$ in materials) and outsourcing its manufacturing (~300$ for our first prototype). Use 6061 aluminum.
Enclosure, 3D printed				1	2	2	4	Price is approximate; depends on cost of filament and machine use (if any)
				**Total:**		**200.825**	**401.65**	

Tubing and fittings (non-exhaustive)

The connections are, roughly: Pump -> 1/4" ID tubing -> 1/4"-1/8" reducer -> 1/8" ID tubing -> Moisture filter -> check valve -> (Y-splitter if using several regulators) -> pressure regulator(s) -> Valve manifolds. Each valve outlet is connected with 3/32" ID tubing to luer lock connectors -> stainless steel needles (20 gauge) -> 1/32" ID tubing -> needles -> chip.

Luer lock connectors are recommended as a way to quickly connect and disconnect tubing. They are suitable for the pressures used here (< 35 psi (2.4 atm))

Description	Supplier	Supplier part number	Quantity	Unit price (USD)	Extended price (USD)	Notes
Luer lock coupling, male, for 3/32" tubing. Pack of 10	McMaster-Carr	51525K122	4	4.13	16.52	
Luer lock coupling, male, for 1/8" tubing. Pack of 10	McMaster-Carr	51525K123	1	4.13	4.13	
Luer lock coupling, female, for 1/8" tubing. Pack of 10	McMaster-Carr	51525K213	1	3.67	3.67	
Luer lock coupling, male, for 1/4" tubing. Pack of 10	McMaster-Carr	51525K126	1	4.13	4.13	
Luer lock coupling, female, for 1/4" tubing. Pack of 10	McMaster-Carr	51525K216	1	4.03	4.03	
Stainless Steel Dispensing Needle with Luer Lock Connection, 20 gauge. Pack of 50	McMaster-Carr	75165A677	1	12.15	12.15	20 gauge = 0.026" ID / 0.036" OD. (0.66/0.91mm). These are used to connect the valves to the 1/32" tubing; additionally, the needle part of these can be extracted easily (with pliars) and used to connect the 1/32" tubing to the chip.
Plastic Barbed Tube Fitting, Y connector, for 1/8" ID tubing. Pack of 10	McMaster-Carr	5463K118	1	7.37	7.37	Splitter between pump and two pressure controllers
Tygon tubing, firm. 3/32" ID, 5/32" OD. 10ft	McMaster-Carr	5239K25	1	11.7	11.7	For valve outlets. Place male luer lock connectors on other side.
Tygon tubing, soft. 1/32" ID, 3/32" OD. 50ft.	McMaster-Carr	5155T11	1	12.5	12.5	Valves -> chip
PTFE tubing, 25ft. 1/32" ID, 1/16" OD	McMaster-Carr	5239K23	1	25	25	Valves -> chip. PTFE tubing is recommended for use with cells; otherwise, use tygon which is cheaper and more flexible (easier to work with).
Tygon tubing, firm. 1/8" ID, 1/4" OD. 10ft.	McMaster-Carr	5103K32	1	14.8	14.8	Pumps -> Pressure regulators -> Manifolds. Firm tubing is recommended to avoid leaks.
Tygon tubing, firm. 1/4" ID, 3/8" OD. 5ft.	McMaster-Carr	5103K34	1	9.15	9.15	
Thread-to-barb fittings, 10-32 UNF to 3/32" barb. 25/pack	Pneumadyne	EB25	2	14	28	For valve outlets
Thread-to-barb fittings, 10-32 UNF to 1/8" barb. 25/pack	Pneumadyne	EB30	1	14	14	For pressure regulator in/out and manifold inputs
10-32 socket set screws, pack of 25	McMaster-Carr	94355A330	1	2.71	2.71	For plugging unused port on pressure regulators
				**Total**	**169.86**	
